# Probabilistic Damage Modeling and Thermal Shock Risk Assessment of UHTCMC Thruster Under Transient Green Propulsion Operation

**DOI:** 10.3390/ma18153600

**Published:** 2025-07-31

**Authors:** Prakhar Jindal, Tamim Doozandeh, Jyoti Botchu

**Affiliations:** Space System Engineering Section, Space Engineering, Faculty of Aerospace Engineering, TU Delft, Kluyverweg 1, 2629 HS Delft, The Netherlands; tamimdoozandeh@gmail.com (T.D.); b.v.s.jyoti@tudelft.nl (J.B.)

**Keywords:** thermal shock analysis, green bipropellant thrusters, stress margin and fatigue risk, probabilistic damage modeling, rocket nozzle design, ceramics, UHTCMCs

## Abstract

This study presents a simulation-based damage modeling and fatigue risk assessment of a reusable ceramic matrix composite thruster designed for short-duration, green bipropellant propulsion systems. The thruster is constructed from a fiber-reinforced ultra-high temperature ceramic matrix composite composed of zirconium diboride, silicon carbide, and carbon fibers. Time-resolved thermal and structural simulations are conducted on a validated thruster geometry to characterize the severity of early-stage thermal shock, stress buildup, and potential degradation pathways. Unlike traditional fatigue studies that rely on empirical fatigue constants or Paris-law-based crack-growth models, this work introduces a simulation-derived stress-margin envelope methodology that incorporates ±20% variability in temperature-dependent material strength, offering a physically grounded yet conservative risk estimate. From this, a normalized risk index is derived to evaluate the likelihood of damage initiation in critical regions over the 0–10 s firing window. The results indicate that the convergent throat region experiences a peak thermal gradient rate of approximately 380 K/s, with the normalized thermal shock index exceeding 43. Stress margins in this region collapse by 2.3 s, while margin loss in the flange curvature appears near 8 s. These findings are mapped into green, yellow, and red risk bands to classify operational safety zones. All the results assume no active cooling, representing conservative operating limits. If regenerative or ablative cooling is implemented, these margins would improve significantly. The framework established here enables a transparent, reproducible methodology for evaluating lifetime safety in ceramic propulsion nozzles and serves as a foundational tool for fatigue-resilient component design in green space engines.

## 1. Introduction

The recent focus on clean and reusable space propulsion systems has amplified the need for thermostructurally robust materials capable of withstanding extreme temperatures, sharp thermal gradients, and repetitive start–stop cycles without significant degradation [1,2,3,4,5]. Ultra-High-Temperature Ceramic Matrix Composites (UHTCMCs), particularly those based on ZrB_2_–SiC reinforced with carbon fibers, have emerged as promising candidates for structural components, such as thruster nozzles, chambers, and flanges, due to their high temperature capability, low density, and damage-tolerant architecture. Fiber-reinforced UHTCMCs have shown not only high mechanical strength at elevated temperatures but also remarkable thermal shock resistance [6,7,8]. Zoli et al. [7] reported that a unidirectional Cf–ZrB_2_ composite exhibited an increase in flexural strength from ~360 MPa at room temperature to over 550 MPa at 1500 °C, maintaining its integrity even after repeated thermal shocks up to 1400 °C, with only ~16% degradation in strength. These behaviors were attributed to a dense matrix, optimized fiber-matrix bonding, and well-controlled thermal expansion mismatch. Further, Zoli and Sciti [9] demonstrated that matrix densification and improved infiltration routes raised the strength and oxidation resistance of such composites at temperatures exceeding 1650 °C, although toughness and erosion behavior varied depending on the bonding architecture. These studies clearly indicate that UHTCMCs can offer low density (~3.7 g/cm^3^), high strength beyond 1500 °C, and intrinsic crack tolerance, attributes essential for high-reliability engine components [9]. NASA’s research found that fiber-reinforced Ultra-High-Temperature Ceramics (UHTCs) have the advantage of being resistant to crack growth and failure compared to monolithic ceramics. These findings suggest UHTCMCs are promising for high-heat structures. NASA highlighted that tailored UHTCs (e.g., HfB_2_/SiC) were also considered for sharp leading edges on hypersonic vehicles, since microstructural control (grain size, additives) can yield materials suitable for >2000 °C TPS components [10].

The suitability of these composites for propulsion systems has also been demonstrated experimentally. Sciti et al. [11] fabricated reusable nozzles using hot-pressing and spark plasma sintering methods and fired them repeatedly in hybrid rocket motors, observing no measurable erosion and good oxidation protection through the formation of surface ZrO_2_ and SiO_2_ layers. Earlier studies showed that ZrB_2_–Cfiber nozzle segments survived over 30 s of jet impingement at ~2730 K and 2.5 MW/m^2^ heat flux in HVOF torches without any structural failure or measurable ablation [12]. These outcomes reinforce the viability of UHTCMCs in transient propulsion environments and confirm their resistance to rapid heating and high-speed reactive gas flows, unlike conventional graphite or C/SiC systems that exhibit rapid ablation under such loads.

While deterministic thermo-structural simulations are effective for preliminary design, they often fail to capture the material variability, uncertainty in loading conditions, and degradation pathways that dictate the real-world lifetime of UHTCMCs. In practice, ceramics like UHTCMCs exhibit strength scatter governed by surface flaws, porosity, and processing routes, making statistical analysis essential for robust failure prediction [13]. NASA’s CARES/Life modeling [14] for ceramic matrix composites incorporates probabilistic frameworks, such as Weibull distributions, slow crack growth (SCG) models, and fracture mechanics-based fatigue estimators, like Paris’ law and its Walker modification [15]. These models describe time-dependent damage accumulation under cyclic or transient loading and allow estimation of survival probability over mission duration, particularly when integrated with uncertainty quantification techniques, such as Monte Carlo simulations [16] or Fast Probability Integration [17]. In particular, probabilistic fatigue analyses for ceramic composites involve combining stress-intensity-based crack growth kinetics (da/dN α ΔK^n^) with statistical strength distributions across populations. Studies using CARES/Life and NASA’s CMC design tools have shown that a single deterministic stress value is insufficient for design; instead, failure probabilities must be computed across safety factor bands (e.g., ±20%) and for expected thermal–mechanical environments [14,18,19]. Such approaches are not yet widely applied to UHTCMC nozzles despite their importance in high-temperature, multi-burn thruster scenarios.

Unlike these approaches, which are often limited by the availability of extensive experimental fatigue data for novel materials like UHTCMCs under extreme transient conditions, this study introduces a novel simulation-derived stress-margin envelope methodology. This method directly assesses the material’s ability to withstand localized stress peaks relative to its temperature-dependent strength, offering a more physically grounded approach for early-stage damage risk assessment in highly transient environments where crack initiation is the primary concern, rather than propagation.

The literature lacks integrated damage risk frameworks tailored for UHTCMCs in green propulsion thrusters, especially under highly transient, short-burst conditions. Specifically, there is a gap in methodologies that can provide a proactive, simulation-based risk assessment for damage initiation without relying on extensive, often unavailable, experimental fatigue data for such unique operating conditions and material systems. Our model directly addresses this by introducing a time-resolved, probabilistic degradation analysis rooted in thermal shock risk and material strength scatter. This work is complementary to classical probabilistic frameworks, such as NASA CARES/Life [14], and draws on insights from prior thermal shock degradation studies by Zoli et al. [7] and recent fatigue reliability evaluations in UHTCMC structures by Reimer et al. [20].

This study builds upon our previous FEA-informed thermo-structural design work [21], where wall thickness optimization and geometric sensitivity analyses were performed using validated transient thermal loads derived from hydrogen-peroxide-based bipropellant simulations. While the earlier work focused on deterministic margin estimation, the current study extends that foundation by integrating time-resolved probabilistic stress envelope modeling, normalized thermal shock metrics, and risk index mapping to predict early-stage failure regions with greater fidelity.

The goal of this work is to develop a simulation-based probabilistic risk assessment framework for evaluating thermal shock sensitivity and early-stage fatigue behavior in UHTCMC thrusters operating under transient green bipropellant conditions. Using a validated finite element model of a ZrB_2_–SiC–Cfiber thruster structure, this study analyzes degradation-sensitive zones, such as the convergent throat, inner flange curvature, and downstream nozzle wall. It incorporates time- and temperature-dependent flexural strength, probabilistic safety margins, and fatigue damage accumulation into a unified risk assessment framework. Multiple operational windows are evaluated, including 0–0.59 s, 0.59–3.0 s, and >3.0 s, to map region-specific failure probabilities. Furthermore, a normalized risk index is defined to quantify the time-resolved proximity to failure across critical axial zones. The presented framework provides practical tools for mission-driven thruster sizing, identifying damage-prone regions for structural reinforcement and determining safe burn durations. This supports lifetime prediction in reusable ceramic thrusters using sustainable propellants. In combination with our earlier optimization findings [21], this offers a dual benefit of structural reliability and mass efficiency for green propulsion systems.

## 2. Materials and Reference Model

This section provides a detailed description of the UHTCMC material system under study, the validated thruster geometry used for thermos-structural simulations, and the underlying mathematical formulations governing the transient stresses, strain, and thermal response. All the results presented in this paper are based on an optimized configuration previously validated through finite element analysis, which is now extended to a probabilistic degradation and failure framework.

### 2.1. Material System: ZrB_2_–SiC–Cfiber UHTCMC

The selected material is a fiber-reinforced UHTCMC comprising a ZrB_2_–SiC matrix reinforced with randomly oriented carbon fiber. This architecture combines the crack resistance and thermal stability of UHTCs with the non-catastrophic failure modes and elevated fracture toughness of ceramic composites. Recent studies [6,8,11,22,23] confirm that such composites maintain high flexural strength above 1500 °C with minimal degradation even after thermal shock cycles and are effective in suppressing erosion in rocket nozzle applications.

The mechanical behavior of the composite is temperature-dependent and incorporates flexural strength degradation, modulus reduction, and thermal expansion variation. Table 1 summarizes the key thermophysical and mechanical properties of the material, adapted from prior studies and databases. Although fiber-reinforced ceramic matrix composites may exhibit anisotropic behavior depending on fiber orientation and layup, the selected material architecture with randomly oriented short carbon fibers enables a reasonable assumption of macroscale isotropy. This assumption simplifies the thermo-mechanical analysis while still capturing the essential degradation and risk characteristics of the material under transient thermal shock conditions. While localized anisotropy could influence micro-scale stress concentrations (especially near curved geometries, such as the convergent throat), its effect on the global stress response and damage envelope is considered minimal for this level of fidelity. Full anisotropic modeling would require detailed experimental orientation data, which is currently unavailable and beyond the present scope.

The temperature-dependent properties (Cp, k) used in Table 1 are adapted from experimental databases and extrapolated where necessary from refs. [6,11,22]. A curve-fitting routine was used to match the reported data within the 300–2000 K range. The flexural strength degradation with temperature is modeled using a second-order polynomial curve fit derived from the validated material dataset, ensuring accuracy in critical failure prediction zones. This fit (Equation (12)) is applied over the full temperature range from 300 K to 2500 K and captures the strength peak and decline behavior with high accuracy (mean error < 3%).

(1) $$\sigma_{f}\left(T\right)=235.05+0.4850\cdot T-0.000221\cdot T^{2}$$

At T = 1100 K, the predicted flexural strength is *σ_f_*(1100) = 501.1 MPa. This value aligns with strength behavior derived from the computational analysis and is implemented in the FEA code through user-defined temperature-dependent input functions.

### 2.2. Reference Geometry and Model Setup

The selected thruster geometry is a validated axisymmetric model of a 100 N bipropellant thruster, previously optimized for minimum stress and mass. The configuration includes three primary zones: A and B, the flange base and combustion chamber (wall thickness tapers from 4 mm to 2 mm), and C, the convergent and divergent nozzle section (constant 2 mm).

**Figure 1:**
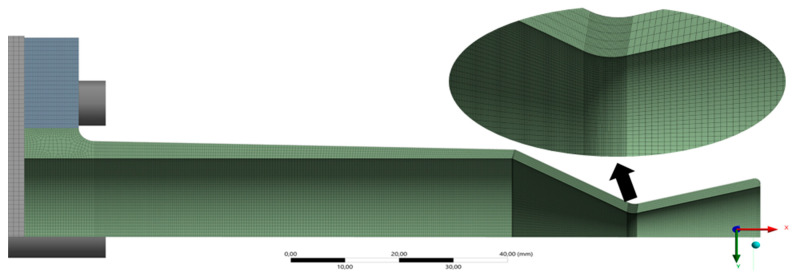
Schematic of the thruster model with the final mesh, along with the zoomed throat section showcasing the custom fine mesh.

The total thruster length is 135.72 mm, with a throat diameter of 9 mm, chamber length of 60 mm, and nozzle divergence half-angle of 15°. A custom meshing strategy was employed to refine regions of expected thermal and mechanical gradients. Figure 1 shows the final meshed model used for all the simulations.

A mesh independence study was performed using four mesh configurations, with convergence validated based on maximum principal stress and peak wall temperature. The results are summarized below in Table 2.

Although M3 and M4 differ by <0.1% in peak stress, M4 was selected for its superior mesh control. It preserves uniform element quality across all wall-thickness variations (e.g., 2 mm to 4 mm configurations), especially near critical curvature zones. This ensures statistical consistency in thermal stress comparisons and probabilistic modeling.

### 2.3. Governing Thermo-Mechanical Equations

The simulation is governed by coupled heat conduction and mechanical equilibrium equations under transient boundary conditions. For an isotropic thermo-elastic material, the governing heat conduction Equation (2) is as follows:

(2) $$\rho C_{p}(T)\frac{\partial T}{\partial t}=\;\nabla \cdot \left(k\nabla T\right)+\;\dot{Q}$$

where *T* is temperature (K), $\dot{Q}$ is the volumetric heat generation rate (assumed zero), *k* is the thermal conductivity, and material properties vary with temperature, as defined above [24,25].

The mechanical equilibrium equation under thermal loading [20,26,27,28] is shown in Equation (3):

(3) ∇· σ+f=0

With the constitutive relationship as shown in Equation (4):

(4) $$\sigma =D(T)\;:(\varepsilon \;-\;\varepsilon_{th})$$

The thermal strain is given by Equation (5):

(5) $$\varepsilon_{th}=\;\alpha \left(T\right)\cdot \;∆T\cdot I$$

where *I* is the identity tensor, and Δ*T = T − T_ref_* is the temperature increment relative to the reference temperature (*T_ref_* = 300 K).

The temperature-dependent elasticity tensor *D*(*T*) for an isotropic material [17,29] is expressed as Equation (6):

(6) $$D\left(T\right)=\frac{E\left(T\right)}{\left(1+\;\nu \right)\left(1-2\nu \right)}\;\times \;\left[\begin{array}{cccccc} 1-\nu & \nu & \nu & 0 & 0 & 0\\ \nu & 1-\nu & \nu & 0 & 0 & 0\\ \nu & \nu & 1-\nu & 0 & 0 & 0\\ 0 & 0 & 0 & \frac{1-2\nu }{2} & 0 & 0\\ 0 & 0 & 0 & 0 & \frac{1-2\nu }{2} & 0\\ 0 & 0 & 0 & 0 & 0 & \frac{1-2\nu }{2} \end{array}\right]$$

For transient simulation, these equations are solved sequentially in time using an implicit solver with user-defined subroutines to incorporate temperature-dependent property updates.

### 2.4. Stress Margin and Damage Model Basis

For failure prediction, the stress margin, *M*(*t*) [30,31], is defined as Equation (7):

(7) $$M\left(t\right)=\;\sigma_{f}\left(T\left(t\right)\right)-\;\sigma_{max}(t)$$

where *σ_f_*(*T*) is the flexural strength at temperature *T*, and *σ_max_*(*t*) is the maximum principal stress at time *t*. A negative *M*(*t*) indicates that local stress exceeds the material strength, signaling potential damage or failure [32]. In probabilistic modeling (Section 4), this margin is treated as a random variable by incorporating scatter in *σ_f_* or *σ_max_* (via variation in Δ*T* or material property). To account for uncertainty in material behavior and operating conditions, this stress margin is treated probabilistically in Section 4. Specifically, a ±20% variability band is applied to *σ_f_*(T), and propagated through *M*(*t*), yielding a statistical envelope for the safety margin.

The ±20% variability band applied to temperature-dependent flexural strength *σ_f_*(T) is based on scatter ranges commonly reported for advanced UHTCMC systems, as seen in the experimental studies by Zoli et al. [7] and Reimer et al. [20], where strength reductions of up to 15–25% were observed due to microstructural flaws, porosity, and fiber misalignment. Additionally, NASA’s CARES/Life methodology [14] recommends applying conservative safety factors in the absence of extensive statistical datasets, especially for brittle ceramic composites. Our chosen variability band thus represents a physically reasonable envelope for capturing the most likely material strength distribution.

**Figure 7:**
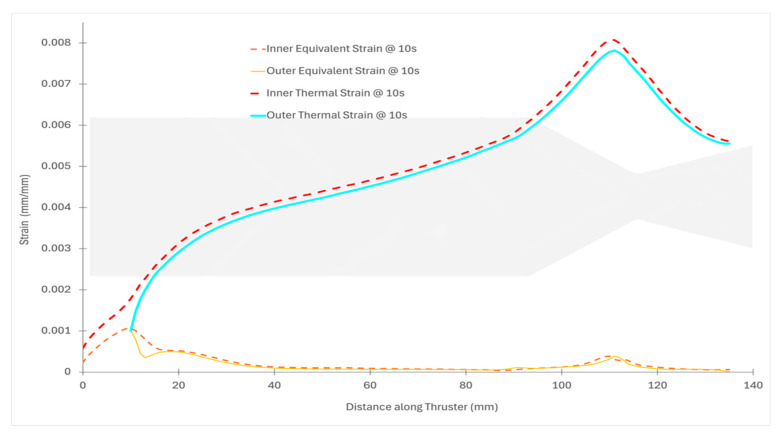
Distribution of strain field across the entire thruster at the peak stress time of 10 s.

Furthermore, this strength range was validated against our earlier transient thermo-structural model of the same UHTCMC thruster geometry [21], which demonstrated convergence in stress and thermal predictions across multiple material assumptions. In this study, the effect of this ±20% variability is directly propagated into the computed stress margin envelopes and risk index, as shown in Figure 7. These envelopes serve as a qualitative sensitivity band, indicating how the local risk classification (green/yellow/red) may shift depending on strength fluctuations, without requiring full Weibull parameterization. This approach ensures a conservative yet practical prediction of early damage zones under transient operation.

### 2.5. Thermal and Mechanical Loading Conditions

The thruster is subjected to a time-dependent heat flux profile and internal chamber pressure over a 10 s firing cycle. Thermal loading is modeled via convective flux applied to the internal surface using Equation (8):

(8) $$q^{\prime \prime }\left(x,\;t\right)=h\left(x,t\right)\cdot [T_{gas}\left(x,t\right)-T_{wall}\left(x,\;t\right)]\;$$

where *h* and *T_gas_* vary along the axial direction and are implemented as spatially dependent boundary functions based on chamber and nozzle flow conditions. The spatial and temporal distributions of the convective heat transfer coefficients and gas-side temperature profiles, which serve as the primary thermal boundary conditions for the FEA model, were directly extracted from prior CFD simulations of H_2_O_2_–kerosene transient firings, as detailed in our previously published work [21]. This CFD-coupled approach ensures realistic transient thermal loading and fidelity to the complex flow phenomena within the thruster chamber and nozzle, particularly during short-duration ignition events. These CFD-derived profiles have previously been benchmarked against expected heat flux behavior in different bipropellant thrusters, lending confidence to the early-time thermal evolution predicted by the FEA. Internal pressure varies with burn profile, peaking at 2.5 MPa in the combustion chamber. The outer walls are adiabatic, and mechanical constraints are applied at the flange face. No active cooling is assumed, representing a worst-case firing profile for assessing failure risk.

## 3. Thermal Shock and Transient Stress Margin Behavior

This section investigates the early-time thermal and mechanical response of the UHTCMC thruster under transient hot-fire loading. The primary aim is to quantify the severity of thermal shock and assess its contribution to early-stage stress buildup in critical structural regions. Thermal gradient rates (Δ*T*/Δ*t*), normalized shock indices, and evolving stress margin envelopes are used to identify degradation-prone zones between 0 and 3 s of operation, particularly across the flange (0–30 mm), chamber (30–90 mm), and nozzle (90–135.72 mm).

### 3.1. Transient Temperature Rise and Gradient Evolution

**Figure 2:**
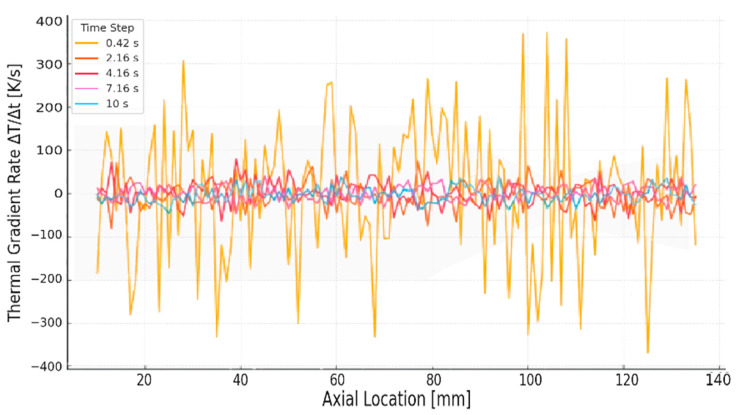
Axial thermal gradient rate across the thruster length.

During the early stages of hot-fire operation, the UHTCMC thruster experiences steep temperature transients driven by convective heat transfer from high-temperature combustion gases (*T_gas_* ≈ 3000 K) [26,33,34,35]. The inner nozzle wall, being thin and directly exposed, heats up more rapidly than the outer wall, resulting in high axial and through-thickness thermal gradients. This thermal imbalance generates expansion-induced strain and localized stresses that govern early-stage material degradation. To visualize this thermal loading rate, Figure 2 plots the spatially resolved axial thermal gradient rate (Δ*T*/Δ*t*) across the thruster length at five critical times: 0.42, 2.16, 4.16, 7.16, and 10 s. The Δ*T*/Δ*t* values were computed using finite differences over measured temperature snapshots from validated FEA thermal simulations, using Equation (9):

(9) $$\frac{\Delta T}{\Delta t}\left(x\right)=\;\frac{T\left(x,\;t_{2}\right)-T(x,\;t_{1})}{t_{2}-\;t_{1}}$$

where *t*_1_ and *t*_2_ represent successive time steps. Each curve reflects the net thermal flux experienced by the material across the wall thickness (inner to outer) at that moment, representing the thermal shock intensity evolving during the transient firing sequence.

At 0.42 s, the thermal gradient rate spikes dramatically near the convergent throat (≈110 mm), reaching +370 K/s on one end and reversing to −380 K/s immediately downstream. This sharp polarity reversal is particularly dangerous, indicating a fast inversion in relative heating rates between the inner and outer walls. It signals maximum thermal shock potential and strain discontinuity at this location. Initially, the convergent throat region (≈110–120 mm) experiences the steepest thermal gradients (Δ*T*/Δ*t* ≈ ±370 K/s), resulting in the highest thermally induced strain rates and corresponding normalized thermal shock indices (*S_th_* ≈ 43). This sharp Δ*T*/Δ*t* peak at *t* ≈ 0.42 s is consistent with and directly reproduced from the transient thermal flux profiles identified in our earlier study [21], where the throat was shown to exhibit intense localized heating due to geometric flow constriction and stagnation effects. By 2.16 s, the gradients are still elevated in the same region (~150–200 K/s), but the profile begins to smooth as heat conduction progresses. At 4.16 s, the peak Δ*T*/Δ*t* reduces further and broadens upstream, indicating a transition to wider-area heating. At 7.16 s and 10 s, gradients across the full length diminish significantly (<80 K/s), and the axial profile flattens, signaling that the structure has entered a quasi-steady thermal diffusion phase.

This figure confirms that the pronounced peak in the thermal gradient rate at early times aligns with locations later identified in Section 3.2, Section 3.3, Section 3.4 as being most susceptible to thermal shock-induced stress accumulation and early fatigue damage. These gradient profiles also form the basis for computing thermal shock index (*S_th_*) and strain-rate-induced stress estimates in subsequent analysis.

To quantify transient thermal shock severity, a normalized thermal shock index, $S_{th}$, is defined using Equation (10). This equation follows the classic Hasselman and Kingery-type [36,37,38] thermal shock resistance model, balancing a material’s cracking resistance (*σ_f_ · λ*) against its thermal stress sensitivity (*E · α · *Δ*T*/Δ*t*). It incorporates transient effects via the thermal gradient rate Δ*T*/Δ*t*, making it particularly relevant for UHTCMC-based thrusters under rapid heating.

(10) $$S_{th}\left(x,\;t\right)=\frac{\sigma f\cdot \lambda }{E\cdot \alpha \cdot (∆T/\Delta t)}\;$$

Using the averaged material properties for the UHTCMC fed in the computational study (*σ_f_* = 422.83 MPa, *λ* = 45.44 W/m·K, *E* = 252.96 GPa, *α* = 5.288 × 10^−6^ K^−1^) and the maximum observed thermal gradient rate across the inner wall (Δ*T*/Δ*t* ≈ 380 K/s), the computed index becomes 43.02.

This value reflects a favorable balance of thermal shock resistance for UHTCMC ceramics. The literature suggests that typical *S_th_* values for advanced ceramics range depend on the specific material properties under aggressive thermal loading [9,13,17,29]. Thus, the *S_th_* index serves not only as a comparative metric but also as a physically grounded parameter that links the operational thermal environment to the intrinsic material behavior. While it does not replace full-field stress or strain analysis, it provides a practical scalar to interpret where shock-prone zones may initiate damage under aggressive startup conditions.

It indicates that the nozzle throat zone (≈110–120 mm) experiences severe transient mismatch, validating the stress localization results observed in FEA and supporting its identification as a critical degradation zone.

Furthermore, the thermally induced strain rate $\dot{\varepsilon_{th}}$ can be estimated using Equation (11):

(11) $$\dot{\varepsilon_{th}}=\;\alpha \left(T\right)\;\cdot \;\frac{∆T}{∆t}$$

For example, at *T* ≈ 1100 K and *α* (1100 K) ≈ 5.72 × 10^−6^/K with maximum observed Δ*T*/Δ*t* ≈ 380 K/s, $\dot{\varepsilon_{th}}$ ≈ 0.00217 s^−1^. This is within the range of strain rates used in thermal fatigue modeling and provides a critical input for stress estimation. The associated thermally induced stress, $\sigma_{th}\approx E\left(T\right)\times \dot{\varepsilon_{th}}$, evaluates to ≈499.1 MPa. This estimated stress closely matches FEA results at the same location and time (discussed in Section 2.2), supporting the validity of this analytical approximation.

All these values confirm that the convergent throat region is the most vulnerable during the first 2–3 s of operation. These observations form the basis for stress margin analysis and probabilistic failure modeling developed in subsequent sections.

### 3.2. Stress Build-Up and Peak Stress Timing

The steep thermal gradients observed during the first few seconds of thruster operation led to significant thermal strain, which induced mechanical stress buildup in structurally constrained regions. To evaluate the evolution and localization of these stresses, the maximum principal stress distribution is analyzed as a function of time and location across the thruster wall.

**Figure 3:**
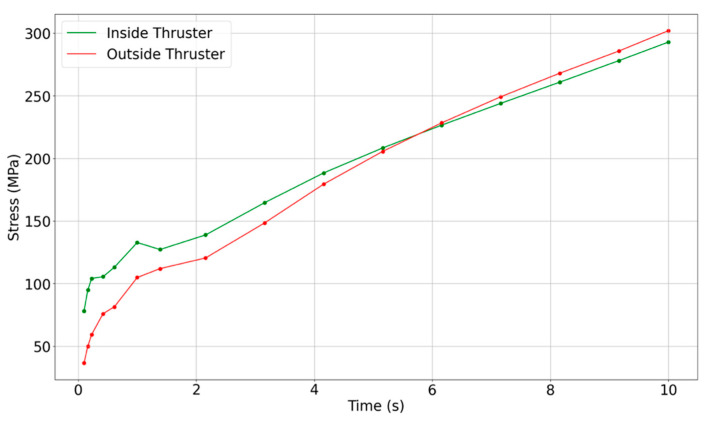
Temporal evolution of maximum principal stress at the inner (green) and outer (red) wall surfaces of the thruster during a 10 s hot-fire simulation. Colors represent structural location. Stress remains below temperature-dependent material failure limits throughout this window.

Figure 3 shows the temporal evolution of maximum principal stress in different axial zones of the thruster over a 10 s simulation window. The stress within the nozzle region peaks at approximately *t* ≈ 2.16 s, with magnitudes approaching 372 MPa. In contrast, the flange region experiences a delayed buildup, reaching its maximum around *t* ≈ 3.68 s. This temporal separation highlights two key physical effects: (i) Since the nozzle wall is thin and directly exposed to hot gas flow, it undergoes rapid thermal expansion generating high tensile stresses early in the burn. (ii) The flange experiences a slower temperature rise due to its greater thickness and insulation, but its rigidity causes strain accumulation that ends in delayed stress concentration.

**Figure 4:**
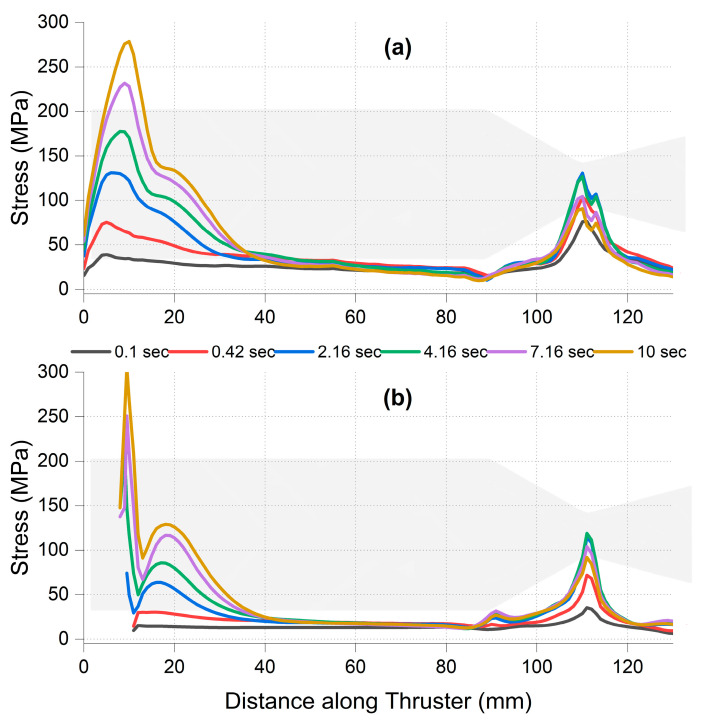
Spatial distribution of maximum principal stress along the thruster length at six critical burn times. Subfigure (**a**) shows the inner wall; (**b**) shows the outer wall. Color-coded traces represent transient snapshots at 0.1, 0.42, 2.16, 4.16, 7.16, and 10 s. Axial position is measured from the flange base toward the nozzle exit.

Figure 4 provides the spatial distribution of maximum principal stress on the inner and outer surfaces along the thruster length for all the firing durations. On the inner surface (Figure 4a), the convergent throat region (~115–118 mm) shows the highest stress magnitudes at the critical time of 2.16 s. This aligns with the peak Δ*T*/Δ*t* and thermal shock index *S_th_* values identified earlier in Section 3.1. Meanwhile, on the outer surface (Figure 4b), stress concentrations are observed at the flange curvature (10–20 mm) and the upstream chamber–nozzle transition zone (~85–90 mm).

The flange–chamber junction (0–30 mm) exhibits alternating tensile and compressive stress zones. These are consistent with stress reversal zones induced by thermal bending and represent potential hotspots for cyclic fatigue and sub-surface cracking. The presence of both sharp curvature and thermal mismatch in this region reinforces its importance for life prediction analysis [28,39,40,41]. These results indicate that while the convergent nozzle throat is the earliest region to reach peak stress, the flange curvature becomes more critical at longer durations due to accumulated thermal strain. This transition in peak stress location over time will directly impact the interpretation of stress margin behavior and damage susceptibility, which is examined in Section 3.1.

### 3.3. Transient Stress Margin Envelopes

To evaluate the structural robustness of the UHTCMC thruster under hot-fire transient loading, it is essential to examine how close the local stresses operate relative to the material’s failure envelope. This is achieved by computing the temperature-dependent stress margin, using Equation (7), at each time step and location, where *σ_f_*(*T*) is derived from Equation (12) to the measured flexural strength of the material across a wide temperature range. To capture the natural variability in ceramic strength, a ±20% uncertainty band is applied around the computed margin values, consistent with the strength scatter observed in experimental studies on UHTCMCs [7,10,42] and probabilistic design frameworks [14,17,32,43].

**Figure 5:**
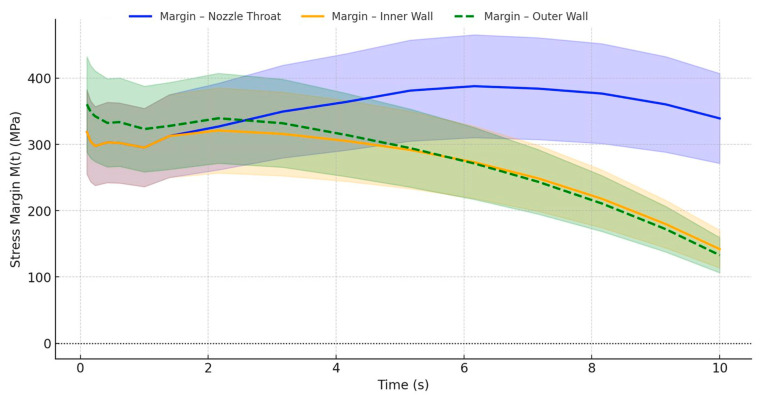
Transient stress margin envelopes with ±20% strength uncertainty.

Figure 5 shows the transient evolution of stress margins, *M*(*t*), at three structurally critical regions: the inner convergent nozzle near the throat, the inner wall near the flange–chamber interface, and the outer flange curvature. Each margin is plotted as a central curve, with shaded bands reflecting ±20% variability in the actual margin (rather than just *σ_f_* (*T*)), offering a more intuitive picture of structural risk over time.

The nozzle throat shows a high initial margin of approximately 319 MPa at 0.1 s, which continues to increase modestly and peaks at 339 MPa around 10 s. Contrary to general assumptions of margin collapse, this region remains structurally robust throughout the 10 s operation, maintaining a margin well above 300 MPa. The inner wall exhibits a gradual decline in margin from 319 MPa to 142 MPa by 10 s, with the critical threshold of 150 MPa being crossed just before the 10 s mark, indicating the onset of structural concern only at prolonged durations. In contrast, the outer wall margin steadily reduces from about 361 MPa at 0.1 s to approximately 133 MPa at 10 s. Although it does not drop below zero, this continuous decline highlights a progressive approach toward the material’s limit and supports predictions of cumulative degradation.

Initially, all three regions operate safely with over 300 MPa of stress margin. However, the envelope profiles reveal that none of the regions experience early critical failure, but the inner and outer walls progressively become structurally vulnerable past 8–10 s. These temporal trends not only validate the importance of spatial thermal mismatch but also motivate the use of fatigue modeling and degradation accumulation strategies explored in the next section.

### 3.4. Structural Strain and Deflection Behavior

While stress margins quantify the likelihood of material failure, the evolution of strain and global deflection offers additional insight into deformation mechanics and the long-term durability of the thruster. In this subsection, the thermal strain profiles and deflection maps are analyzed to assess how much the UHTCMC nozzle structure deforms under high thermal gradients and mechanical loads over time.

**Figure 6:**
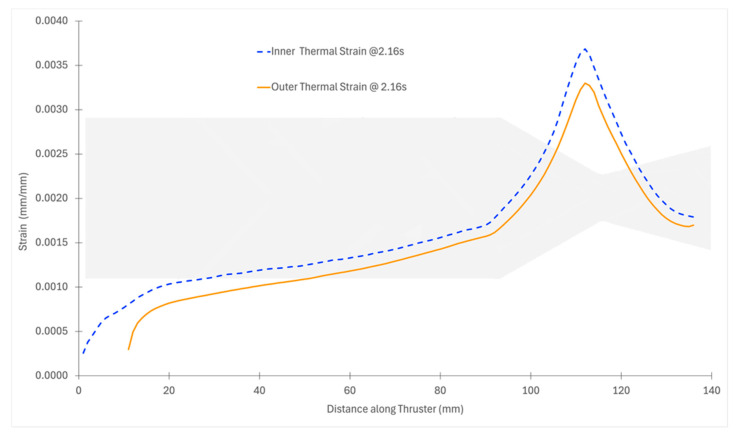
Distribution of strain field across the entire thruster at the peak stress time of 2.16 s.

Figure 6 presents the distribution of maximum thermal strain (*ε_th_*) across the entire thruster at the peak stress time of 2.16 s.

It clearly shows the highest strain concentrations occurring in the inner convergent throat region, where the combination of steep thermal ramp rates and thinner wall geometry amplifies local deformation. The strain values in this region are over seven times greater than those in the upstream flange section, affirming the earlier findings of stress vulnerability.

Figure 7 compares the strain field at 10 s, revealing how thermal strain magnitudes have evolved. At this time step, the peak nozzle thermal strain reaches nearly 16 times that of the flange’s equivalent strain. The expanded strain gap between the nozzle and flange is attributed to the reduced stiffness (*E*(*T*)) at elevated temperatures in the nozzle, leading to larger deformation under the same thermal loading. The Young’s modulus degrades more rapidly with temperature than the increase in thermal strain, causing thermal stresses to decrease while thermal strain increases significantly. Even in the flange region, which is relatively protected, the strain amplifies by more than 3.5 times from the initial cycle.

**Figure 8:**
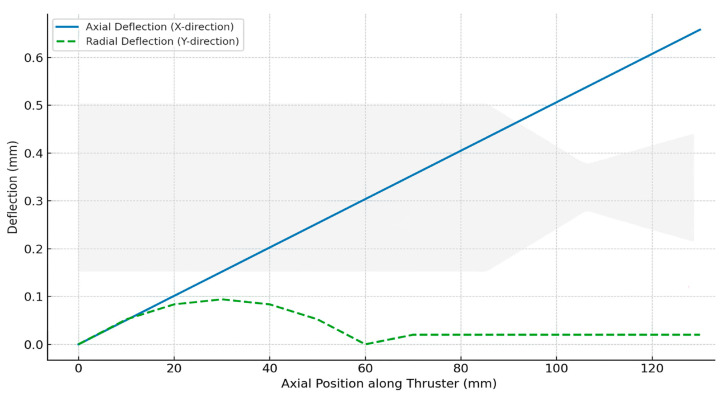
Axial and radial deflection profiles after 10 s of operation.

Figure 8 illustrates the axial and radial deflection profiles of the UHTCMC thruster wall after 10 s of continuous operation. The axial deflection, corresponding to displacement along the thrust direction (*X*-*axis*), exhibits a nearly linear rise from the flange (0 mm) to the nozzle exit, culminating at approximately 0.687 mm. This maximum corresponds to a 0.51% extension relative to the thruster’s total length (135.72 mm) and is primarily attributed to thermal expansion driven by sustained high-temperature loading. In contrast, the radial deflection (*Y*-*direction*) peaks in the vicinity of the flange–nozzle transition zone, around 30 mm from the flange.

Here, the local curvature combined with asymmetric thermal gradients results in a maximum outward displacement of ~0.094 mm. Beyond this point, the radial deflection diminishes as the geometry straightens, and the thermal loading becomes more evenly distributed along the nozzle wall. These deformation profiles not only confirm the spatially varying thermal–mechanical response of the thruster but also validate the previously identified critical zones, namely, the curved chamber–flange region and convergent throat, as sites of potential long-term fatigue accumulation or geometric instability during repeated ignition cycles noted earlier in Figure 4 and Figure 5.

Importantly, these deflections, though measurable, remain within allowable tolerance limits for short-duration thruster burns. The axial elongation and radial deformation are expected to be primarily elastic and recoverable under such thermal–mechanical cycles, assuming no cyclic degradation is introduced. The observed deformation patterns further confirm the convergent throat as a mechanically active site requiring close monitoring in long-term cyclic use or in designs involving multiple re-ignitions.

### 3.5. Cumulative Damage and Degradation Mapping

While early transient stress and strain behavior identify critical structural risk initiation points, predicting cumulative degradation requires tracking how stress margins evolve over operation time and where crack initiation is most likely to occur. In this section, a map of degradation risk based on transient stress margin collapse, spatial stress distributions, and fatigue-sensitive zones is constructed. The goal is to pinpoint failure-prone locations as a function of time, enabling mission-driven risk planning and future probabilistic fatigue modeling.

As shown in Figure 5, the inner convergent throat (located between *x* ≈ 105 and 120 mm) maintains the lowest margin during early operation and comes closest to reaching failure conditions during the first 2–3 s. However, failure initiation does not depend solely on a single-point margin collapse; it also depends on the duration over which a region operates under sub-critical margins, and how localized strain and thermal loading evolve spatially.

**Figure 9:**
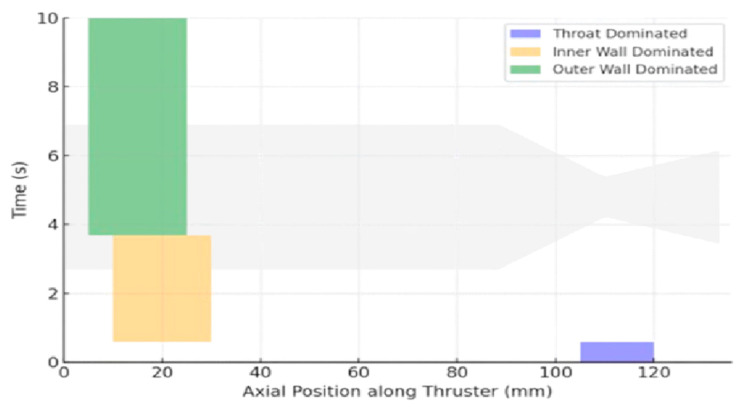
Degradation hotspot evolution across time and thruster length.

To visualize this, Figure 9 presents a schematic degradation map. It shows the most critical degradation zone over time for three distinct operating windows: 0–0.59 s, 0.59–3.68 s, and beyond 3.68 s. These time windows are defined based on the relative margin evolution in the convergent throat, chamber–flange interface, and outer flange curvature. From 0 to 0.59 s, the highest thermal gradient rate and shock index occur near the convergent nozzle throat. This makes the throat region the most critical during short-duration burns, typical of single-pulse green bipropellant firings. Between 0.59 and 3.68 s, degradation risk migrates upstream to the inner wall near the chamber–flange interface (*x* ≈ 10–30 mm), where structural rigidity and geometric constraints amplify stress buildup over time. Beyond 3.68 s, the outer flange curvature (*x* ≈ 5–25 mm) becomes dominant in risk contribution. This region accumulates bending-induced strain due to slower thermal dissipation and material constraint.

This map confirms that thermal–mechanical fatigue in UHTCMC thrusters evolves in a spatially nonuniform and temporally staged manner. No single region dominates risk across the entire burn. Instead, different structural zones rise in fatigue sensitivity depending on pulse duration, with the throat dominating <1 s burns and the flange region governing risks for longer sustained operations.

It is also important to note that this analysis assumes a hot-fire simulation without any active cooling. The lack of wall cooling exaggerates thermal gradients and represents a worst-case scenario. In practice, cooling would suppress thermal gradients, reduce material temperature, and delay margin collapse. As such, the observed degradation windows represent a worst-case envelope, suitable for conservative design screening and mission envelope bounding.

## 4. Probabilistic Damage Modeling and Fatigue Outlook

This section presents a simulation-based fatigue risk analysis derived from the time-resolved stress margin envelopes (Figure 5), thermal strain and deflection behavior (Figure 6, Figure 7 and Figure 8), and the spatial degradation migration mapped in Figure 9. Rather than relying on empirical fatigue-life models (e.g., Paris’ Law [44] or Miner’s Rule [45]), margin collapse dynamics, thermomechanical strain rate indicators, and material strength variability are employed to assess damage-prone regions across the UHTCMC thruster during transient thermal loading. This approach yields a conservative but physically grounded assessment of failure likelihood under repeated pulsed firing conditions.

The temperature-dependent stress margin, *M*(*t*), is the main basis for this analysis at three critical locations: the inner convergent throat (~115 mm), the inner flange–chamber interface (~15 mm), and the outer flange curvature (~20 mm). The margin collapse profiles shown in Figure 5 include ±20% variability in the material’s flexural strength, capturing strength scatter typical of fiber-reinforced UHTCMCs. From these trends, temporal windows are extracted that distinguish which regions dominate degradation at different stages of operation.

**Figure 10:**
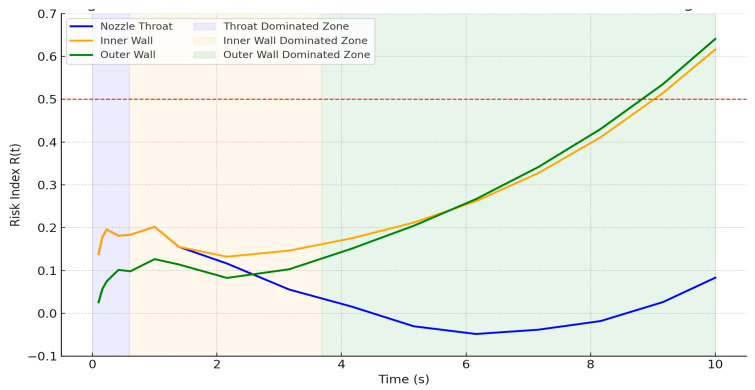
Evolution of risk index in critical zones based on stress margin collapse.

Figure 10 illustrates the evolution of the normalized risk index, defined using Equation (12):

(12) $$R\left(t\right)=1-\;\frac{M(t)}{\sigma_{f0}}$$

where *σ_f_*_0_ = 370 MPa represents the nominal flexural strength at room temperature. It is formulated to provide a first-order approximation of material degradation by translating stress margin depletion into a 0–1 scale. *Note: While the stress margin M*(*t*) *dynamically incorporates temperature-dependent flexural strength, σ_f_*(*T*), *the normalization is performed using room-temperature strength, σ_f_*_0_*, to preserve a consistent baseline across all time steps and spatial locations. This modeling choice avoids double-counting temperature effects already embedded in M*(*t*) *and provides a conservative estimate of progressive margin depletion relative to the material’s most favorable condition.* This substitute metric enables fatigue-sensitive zoning even in the absence of empirical crack propagation data. The shaded bands indicate the dominant degradation zones based on stress margin ranking rather than just absolute stress magnitude.

The nozzle throat exhibits the steepest margin depletion due to intense early-time thermal shock at 0–0.59 s, making it the most critical failure site for short-duration pulses. As stress continues to accumulate, the inner wall near the flange interface overtakes the throat in structural sensitivity during 0.59–3.68 s. This zone becomes critical for medium-duration operation, especially under repeated cycling. The outer flange curvature experiences the slowest degradation initially but becomes the dominant risk region during prolonged thermal exposure (3.68–10 s), as stress gradually localizes due to constraint buildup and poor heat dissipation. This representation preserves the physical interpretation of margin-based failure evolution and agrees with FEA predictions and material degradation physics.

To further consolidate these insights into a spatial–temporal fatigue outlook, Table 3 summarizes the important findings across five major structural regions. Each entry includes the approximate axial location, dominant time window of structural concern, estimated stress margin collapse timing (based on ±20% strength bounds), and a quantitative classification of fatigue risk using both normalized risk index ranges and corresponding stress margin thresholds *M*(*t*). These thresholds were derived based on observed simulation data (Figure 3 and Figure 4), prior ceramic failure behavior [7,14,20], and probabilistic design heuristics. The classification categories (Safe, Moderate Risk, and High Risk) are directly mapped to values of the normalized risk index (0.0–1.0) and *M*(*t*) bands (<200 MPa, 200–300 MPa, >300 MPa), as shown in Table 3.

This tabular mapping serves as a direct engineering tool to assess the susceptibility of various segments under realistic mission profiles. The combination of stress margin envelopes, deformation behavior, and simulated strength variability creates a fatigue-informed degradation map without the need for experimental crack-growth constants or full stochastic field modeling. Moreover, the sequential shift in high-risk zones illustrated schematically in Figure 9 is now fully supported by the time-resolved margin collapse profiles and risk index plots presented in Figure 10. These results show how the dominant risk region transitions from the convergent throat (<0.6 s) to the inner wall (0.6–3.7 s) and eventually to the outer flange beyond 3.7 s. This temporal mapping enables predictive identification of when and where thermal–mechanical degradation is most likely to initiate, which can inform thruster lifetime planning, burst duration constraints, and structural design optimization. While this framework does not yet account for cyclic stress reversal or material fatigue thresholds in terms of *N_f_* (cycles to failure), the presented approach serves as a foundation for fatigue-aware thruster sizing and mission planning in space propulsion applications. Future work may incorporate embedded sensors, in situ monitoring of crack formation, and material-level damage diagnostics to further calibrate this probabilistic outlook [32,46].

## 5. Discussion

This study presents a thermomechanically informed risk framework for UHTCMC thrusters subjected to short-duration green propulsion firing. Unlike traditional deterministic analyses, this work introduces a simulation-derived stress-margin envelope methodology that does not rely on empirical crack growth constants or Paris’ law-type models. Instead, it uses time-resolved margin depletion behavior tied to temperature-dependent strength and probabilistic scatter, offering a first-principles approach to failure risk. A crucial insight is the spatiotemporal migration of damage risk across the thruster structure. Initially, the convergent throat (≈110–120 mm) experiences the steepest thermal gradients (Δ*T*/Δ*t* ≈ ±370 K/s), resulting in the highest thermally induced strain rates and normalized shock indices (*S_th_* ≈ 43). Although this value is lower than the literature estimates, it still identifies this zone as the earliest to experience a significant mismatch in inner-to-outer wall expansion, validating the localized stress concentrations and deformation patterns observed in FEA. Importantly, the analysis shows that thermal shock, rather than absolute temperature, is the dominant driver of early-time degradation, highlighting the need for startup-specific risk control in nozzle design.

Although this study intentionally models a worst-case uncooled thruster configuration, simplified scaling estimates suggest that incorporating active thermal management could significantly improve thermal stress performance. Regenerative cooling channels or ablative liners are known to reduce wall heating rates by approximately 30–50% in similar systems [42], which would correspondingly reduce the axial thermal gradient rate (∆T/∆t) to ~190–250 k/s. According to Equation (10), such a reduction would improve the thermal shock index (*S_th_*) by roughly 40–60%, potentially raising it from ~43 to ~70. This implies a substantial gain in structural resilience against transient damage, reinforcing that the current results present a conservative risk scenario.

As firing progresses beyond ~2 s, heat diffusion reduces gradient asymmetry, but stress migration toward the flange–chamber interface emerges as a second-phase risk mechanism. This transition is driven by accumulated thermal strain, material softening, and geometric constraints that amplify localized bending and tensile stress. The delayed but progressive margin depletion in this region, particularly at the outer flange curvature, points to fatigue-like behavior even within a single firing pulse. The probabilistic treatment of stress margins, through ±20% strength variability, translates simulation outputs into actionable risk maps. The normalized risk index (*R*(*t*)) captures not only the time of margin collapse but also its proximity and severity. Notably, even in worst-case adiabatic conditions, no region drops below critical margins in the first 2 s, implying that conservative mission envelopes could prioritize single-pulse burns under this duration to avoid cumulative degradation.

From a design standpoint, the proposed framework fills a critical gap in the current literature by enabling damage risk mapping for brittle composite thrusters under rapid, short-duration transients, conditions often excluded from fatigue-focused empirical models. These findings suggest that thermostructural resilience in UHTCMC thrusters cannot be captured by static peak stress limits alone. Instead, strain-rate sensitivity, stress gradient reversals, and time-evolving margin envelopes must be considered jointly. The use of scalar indices, like *S_th_*, although not a substitute for full FEA, is shown to correlate with critical locations of strain concentration and provides a compact indicator of thermal mismatch severity. Moreover, the deformation behavior observed (e.g., localized strain amplification and wall bending) underscores the need for stress redistribution mechanisms, such as passive cooling or structural tapering near the flange–nozzle transition.

Overall, this work provides early-stage design tools for mission planners and propulsion engineers to define safe burn durations, strengthen risk-prone zones, and support reusability in ceramic propulsion systems. It reinforces the potential of fatigue-informed probabilistic design for UHTCMCs operating under rapid thermal cycling. The presented framework bridges analytical modeling, FEA, and stochastic variability without relying on empirical crack-growth laws. This provides a scalable path toward certification of ceramic propulsion components for multi-pulse or re-ignitable missions, a growing requirement in low-emission, reusable propulsion systems.

## 6. Conclusions

This work presents a physically grounded, simulation-based framework for assessing fatigue risk and degradation pathways in fiber-reinforced UHTCMC thrusters exposed to transient hot-fire operation. Using validated finite element analysis and stress margin modeling, this study identifies key zones of thermal shock susceptibility, stress buildup, and fatigue-prone behavior. The margin envelope method, combined with conservative material variability, enables a physically justified classification of degradation zones over time. Unlike prior methods that rely on crack-growth calibration or empirical fatigue models, this study introduces a deterministic–probabilistic hybrid tool that can guide safe operating envelope selection and design margin sizing for brittle ceramic structures. All the analyses assumed no active cooling; real-world nozzle cooling would increase safety margins significantly. Following are the major outcomes from this work:

- A maximum Δ*T*/Δ*t* of ~±370 K/s near 110–120 mm at early times (*t* ≈ 0.42 s), signaling peak thermal shock conditions;
- Normalized thermal shock index *S_th_* ≈ 43 under real thermal ramp conditions, consistent with the literature thresholds for strain localization in UHTCMCs;
- All three critical regions, the convergent throat, chamber–flange interface, and outer flange, maintain initial safety margins above 300 MPa, confirming early structural robustness during the first seconds of firing;
- Risk index *R*(*t*) exceeded 0.5 in the outer and inner wall by *t* = 8 s, indicating high susceptibility to failure without cooling;
- Margin collapse at the convergent throat dominates at 0–0.59 s (thermal shock), the chamber–flange interface becomes critical at 0.59–3.68 s (strain accumulation), and the outer flange governs risk beyond 3.68 s (bending-induced fatigue);
- Fatigue risk classification indicates high risk in the inner convergent throat (110–120 mm) within 0.59 s, moderate risk in the chamber–flange interface (10–30 mm) around 8 s, and late-stage transition risk in the outer flange (15–25 mm) beyond 9 s; upstream walls remain in the safe regime throughout.

These findings have practical design implications for reusable green-propellant thrusters. The proposed methodology enables predictive identification of risk-sensitive regions and burn-time thresholds before physical testing, thus reducing material screening costs and accelerating development timelines.

## Figures and Tables

**Table 1:** Temperature-dependent material properties for ZrB_2_–SiC–Cfiber.

Property	Expression/Value	Units
Density, ρ	3.7 × 10^3^	kg/m^3^
Specific heat capacity, Cp	Cp(T) = 500 + 0.1T	J/kg·K
Thermal conductivity, k	k(T) = 26 − 0.008T	W/m·K
Poisson’s ratio, ν	0.18	—

**Table 2:** Mesh convergence study parameters.

Mesh ID	Element Size (mm)	Mesh Type	Total Nodes	T_max_ (K)	σ_max_ (MPa)
M1	1.5	Uniform	97,320	1619.2	369.51
M2	1.0	Uniform	138,420	1633.7	370.86
M3	0.75	Uniform	173,054	1642.7	372.03
M4	0.75	Custom at throat	179,860	1642.7	371.94

**Table 3:** Fatigue risk classification based on stress margin collapse and deformation behavior.

Axial Region	Approx. Location (mm)	Dominant Time Window	Margin Collapse Timing	Likely Damage Driver	Fatigue Risk Classification	Normalized Risk Index Range	M(t) Range (MPa)	Implication
Convergent Throat (Inner)	110–120	0.0–0.59 s	Not below 295 MPa	Thermal shock, early expansion mismatch	High Risk	0.7–1.0	<200	Early failure expected under transient loads
Chamber–Flange Interface	10–30	0.59–3.68 s	t > 8.0 s	Strain accumulation, cyclic loading	Moderate Risk	0.3–0.7	200–300	Sensitive to cyclic effects; progressive degradation likely
Outer Flange Curvature	15–25	3.68–10.0 s	t > 9.0 s	Constraint-induced bending strain	Transition	N/A	N/A	N/A
Nozzle Wall (Outer)	120–130	0.5–1.5 s	No collapse (M > 130 MPa)	Elevated T, low thermal gradient	Safe	0.0–0.3	>300	Stable; low-damage probability during operation
Inner Chamber Wall (Upstream)	40–70	0.0–4.0 s	No collapse	Homogeneous heating, stable margin	Safe	0.0–0.3	>300	

## Data Availability

The original contributions presented in this study are included in the article. Further inquiries can be directed to the corresponding author.

## Nomenclature

**Symbol**	**Description**	**Unit**
*T*(*x*,*t*)	Local wall temperature as a function of position *x* and time *t*	K
*t*	Time during firing sequence	s
*x*	Axial location along the thruster centerline	mm
*ρ*	Density of the UHTCMC material	kg/m^3^
*C_p_*(*T*)	Temperature-dependent specific heat capacity	J/kg·K
*k*(*T*)	Temperature-dependent thermal conductivity	W/m·K
$\dot{Q}$	Volumetric heat generation rate (assumed zero in this study)	W/m^3^
*q*″(*x,t*)	Heat flux at surface due to convection	W/m^2^
*h*(*t*)	Time-dependent convective heat transfer coefficient	W/m^2^·K
*T_gas_*(*t*)	Estimated combustion gas temperature	K
*T_ref_*	Reference temperature for strain calculations (usually 300 K)	K
Δ*T*	Temperature rise relative to reference, *T* − *T_ref_*	K
Δ*T*/Δ*t*	Thermal gradient rate over time	K/s
*α*(*T*)	Coefficient of thermal expansion, linear with *T*	1/K
*ε_th_*	Thermal strain tensor	—
$\dot{\varepsilon_{th}}$	Thermal strain rate	s^−1^
*σ*(*t*)	Cauchy stress tensor at time *t*	MPa
*σ_max_*(*t*)	Maximum principal stress at time *t*	MPa
*σ_f_*(*T*)	Temperature-dependent flexural strength	MPa
*σ_f_*_0_	Nominal flexural strength at room temperature	MPa
*M*(*t*)	Stress margin at time *t*	MPa
*R*(*t*)	Normalized risk index	—
*D*(*T*)	Elasticity tensor (temperature-dependent) for isotropic material	MPa
*E*(*T*)	Temperature-dependent Young’s modulus	GPa
*E*_0_	Young’s modulus at room temperature	GPa
*β*	Modulus degradation factor with temperature	GPa/K
*ν*	Poisson’s ratio	—
*I*	Identity tensor (used in isotropic strain formulation)	—
*S_th_*(*x*,*t*)	Normalized thermal shock index	s^−1^
